# The Multi-Domain Intervention Trial in Older Adults With Diabetes Mellitus for Prevention of Dementia in Japan: Study Protocol for a Multi-Center, Randomized, 18-Month Controlled Trial

**DOI:** 10.3389/fnagi.2021.680341

**Published:** 2021-07-12

**Authors:** Taiki Sugimoto, Atsushi Araki, Hiroki Fujita, Keiko Honda, Nobuya Inagaki, Takeshi Ishida, Junichi Kato, Minoru Kishi, Kazuki Kobayashi, Kunichi Kouyama, Hisashi Noma, Mitsuru Ohishi, Noriko Satoh-Asahara, Hiroyuki Shimada, Kazuhiro Sugimoto, Susumu Suzuki, Yasushi Takeya, Yoshiaki Tamura, Haruhiko Tokuda, Hiroyuki Umegaki, Hirotaka Watada, Yuichiro Yamada, Takashi Sakurai

**Affiliations:** ^1^Center for Comprehensive Care and Research on Memory Disorders, National Center for Geriatrics and Gerontology, Obu, Japan; ^2^Medical Genome Center, National Center for Geriatrics and Gerontology, Obu, Japan; ^3^Department of Diabetes, Metabolism, and Endocrinology, Tokyo Metropolitan Geriatric Hospital, Tokyo, Japan; ^4^Department of Endocrinology, Diabetes and Geriatric Medicine, Akita University Graduate School of Medicine, Akita, Japan; ^5^Department of Medical Nutrition, Kagawa Nutrition University, Saitama, Japan; ^6^Department of Diabetes, Endocrinology and Nutrition, Kyoto University Graduate School of Medicine, Kyoto, Japan; ^7^Department of Internal Medicine, Saitama Citizens Medical Center, Saitama, Japan; ^8^Department of Internal Medicine, Hyogo Prefectural Rehabilitation Center at Nishi-Harima, Tatsuno, Japan; ^9^Department of Internal Medicine, Nishiwaki Municipal Hospital, Nishiwaki, Japan; ^10^Department of Diabetes and Metabolic Disease, Asahi General Hospital, Chiba, Japan; ^11^Department of Diabetes Medicine, Hyogo-Chuo National Hospital, Sanda, Japan; ^12^Department of Data Science, Institute of Statistical Mathematics, Tokyo, Japan; ^13^Department of Cardiovascular Medicine and Hypertension, Graduate School of Medical and Dental Sciences, Kagoshima University, Kagoshima, Japan; ^14^Department of Endocrinology, Metabolism, and Hypertension Research, Clinical Research Institute National Hospital Organization, Kyoto Medical Center, Kyoto, Japan; ^15^Center for Gerontology and Social Science, National Center for Geriatrics and Gerontology, Obu, Japan; ^16^Diabetes Center, Ohta Nishinouchi Hospital, Koriyama, Japan; ^17^Department of Geriatric and General Medicine, Osaka University Graduate School of Medicine, Suita, Japan; ^18^Department of Clinical Laboratory, National Center for Geriatrics and Gerontology, Obu, Japan; ^19^Department of Community Healthcare and Geriatrics, Nagoya University Graduate School of Medicine, Nagoya, Japan; ^20^Department of Metabolism and Endocrinology, Juntendo University Graduate School of Medicine, Tokyo, Japan; ^21^Department of Cognition and Behavior Science, Nagoya University Graduate School of Medicine, Nagoya, Japan

**Keywords:** diabetes mellitus, cognitive decline, dementia, multi-domain intervention, physical exercise, nutrition, social participation, randomized control trial

## Abstract

**Background:** The Japan-Multi-domain Intervention Trial for Prevention of Dementia in Older Adults with Diabetes (J-MIND-Diabetes) is an 18-month, multi-centered, open-labeled, randomized controlled trial designed to identify whether multi-domain intervention targeting modifiable risk factors for dementia could prevent the progression of cognitive decline among older adults with type 2 diabetes mellitus (T2DM). This manuscript describes the study protocol for the J-MIND-Diabetes trial.

**Materials and Methods:** Subjects of this trial will comprise a total of 300 T2DM outpatients aged 70–85 years with mild cognitive impairment. Subjects will be centrally randomized into intervention and control groups at a 1:1 allocation ratio using the stratified permuted-block randomization methods. The intervention group will participate in multi-domain intervention programs aimed at: (1) management of metabolic and vascular risk factors; (2) physical exercise and self-monitoring of physical activity; (3) nutritional guidance; and (4) social participation. The control group will receive usual T2DM care and general instructions on dementia prevention. The primary and secondary outcomes will be assessed at baseline, at 6- and 18-month follow-up. The primary outcome is change from baseline at 18 months in a global composite score combining several neuropsychological domains, including global cognitive function, memory, attention, executive function, processing speed and language. Secondary outcomes include: (1) cognitive changes in neuropsychological tests; (2) changes in geriatrics assessments; (3) metabolic control and diabetic complications; (4) changes in blood and urinary markers.

**Discussion:** This trial will be the first trial to demonstrate the effectiveness of multi-domain intervention in preventing cognitive decline in older adults with T2DM at increased risk of dementia in Japan.

**Trial Registration:** UMIN000035911; Registered on the University Hospital Medical Information Network Clinical Trials Registry (UMIN-CTR) 18 February 2019. (https://upload.umin.ac.jp/cgi-open-bin/ctr_e/ctr_view.cgi?recptno=R000040908).

## Introduction

The incidence and prevalence of dementia have been increasing worldwide. The number of people living with dementia is estimated at 46.8 million in 2015, and expected to increase to 131.5 million by 2050 (Prince et al., 2015).

To date, risk factors for dementia that are potentially modifiable throughout lifespan have been reported (Livingston et al., 2020), which include: less education in early life; hearing loss, traumatic brain injury, hypertension, excessive alcohol consumption and obesity in midlife; and smoking, depression, social isolation, physical inactivity, diabetes and air pollution in later life. Among these, ample evidence suggests that type 2 diabetes mellitus (T2DM) is among the important potentially modifiable risk factors. A meta-analysis demonstrated that patients with T2DM are placed at higher risk of incident mild cognitive impairment (MCI), Alzheimer's disease (AD), and vascular dementia (VaD) than those without (Cheng et al., 2012). While multiple underlying mechanisms may account for the significant link between T2DM and dementia, of these, poor glycemic control, hypoglycemia, glycemic variability, and insulin resistance may be of particular interest, in that they may contribute to neurodegenerative pathologies and cerebrovascular disease (Biessels et al., 2006; Strachan et al., 2011). Thus, it remains an urgent priority to formulate successful strategies against cognitive decline and dementia in older adults with T2DM.

Several randomized controlled trials were conducted in this population with a focus on the effects of intensive glycemic control on cognitive function (de Galan et al., 2009; Launer et al., 2011; Koekkoek et al., 2012). However, these studies have failed to provide evidence to support the beneficial effects of intensive glycemic control on cognition, while one study showed the beneficial effect on total brain volume (Launer et al., 2011). Moreover, it is suggested that severe hypoglycemia associated with intensive glycemic control may rather increase the risk of not only cognitive impairment and dementia but cardiovascular events and mortality (Yaffe et al., 2013; Mattishent and Loke, 2016a,b). In light of the available evidence, therefore, the “Glycemic Targets for Elderly Patients with Diabetes” were put forth in 2017 by the Japan Diabetes Society (JDS)/Japan Geriatrics Society (JGS) Joint Committee (Japan Diabetes Society (JDS)/Japan Geriatrics Society (JGS) Joint Committee on Improving Care for Elderly Patients with Diabetes, 2017), which proposed, along with other guidelines/position statements that to achieve appropriate glycemic control and prevent complications of diabetes, care should be exercised to avoid severe hypoglycemia, specifying lower limits for proposed glycemic targets among older adults, particularly those at high risk (i.e., those who have frailty, cognitive impairment, or dementia) (Sinclair et al., 2011, 2018; Dunning et al., 2014; Japan Diabetes Society (JDS)/Japan Geriatrics Society (JGS) Joint Committee on Improving Care for Elderly Patients with Diabetes, 2017).

Physical exercise and diet are first recommended in the management of T2DM, given that there are numerous evidences to demonstrate that physical exercise, including aerobic and resistance training, improves glycemic control and insulin sensitivity (Willey and Singh, 2003; Way et al., 2016). However, the effects of physical exercise on cognition are shown to be inconsistent among older adults with T2DM (Zhao et al., 2018). In a small randomized controlled trial (RCT), Baker et al. demonstrated that aerobic exercise, continued over 6 months, improved executive function among 28 older adults with glucose intolerance (Baker et al., 2010). Recently, an exploratory analysis from the Lifestyle Interventions and Independence for Elders trial demonstrated that physical activity interventions may benefit cognitive function in older adults with diabetes, but not in those without (Espeland et al., 2017). These results indicate that older adults with T2DM are more likely to benefit from physical exercise interventions, thus suggesting the need for further intervention trials.

In older adults with T2DM, dietary intake and nutritional status are also associated with cognitive decline and incident dementia. The Japanese Elderly Diabetes Intervention Trial, which is a longitudinal study, showed that low intakes of carotene, vitamin B2, pantothenate, calcium and green vegetables predicted cognitive decline among male older adults with diabetes during a 6-year follow up (Araki et al., 2017). Nam et al. demonstrated that lower body mass index (<18.5) at baseline and weight loss after diagnosis of T2DM increased risk of incident all-cause of dementia, especially AD, during an average 3.5-year follow up. Weight gain also increased risk of incident all-cause dementia (Nam et al., 2019). Although these observational studies highlight needs of monitoring and interventions on diet and nutritional status for prevention of dementia, there still lacks evidence from a dietary intervention trial.

Again, several large multi-domain prevention trials have provided the evidence that interventions simultaneously targeting multiple risk factors for dementia in older adults, especially in those at increased risk of dementia, may slow cognitive decline and reduce cognitive impairment (Ngandu et al., 2015; Moll van Charante et al., 2016; Andrieu et al., 2017). However, to date, no multi-domain prevention trial for prevention of cognitive decline has been conducted with a focus on older adults with T2DM.

Therefore, the aim of this trial, named the Japan-Multi-domain Intervention Trial for Prevention of Dementia in Older Adults with Diabetes (J-MIND-Diabetes), is to investigate whether multi-domain intervention (consisting of management of metabolic and vascular risk factors, physical exercise and self-monitoring of physical activity, nutritional guidance, and promoting social engagement) may prevent the progression of cognitive decline among T2DM patients with mild cognitive impairment. This manuscript describes the study protocol for the J-MIND-Diabetes trial, and conforms to the Standard Protocol Items: Recommendations for Interventional Trials (SPIRIT) 2013 statement (Supplementary Table 1).

## Methods and Materials

### Study Design and Setting

**Figure 1:**
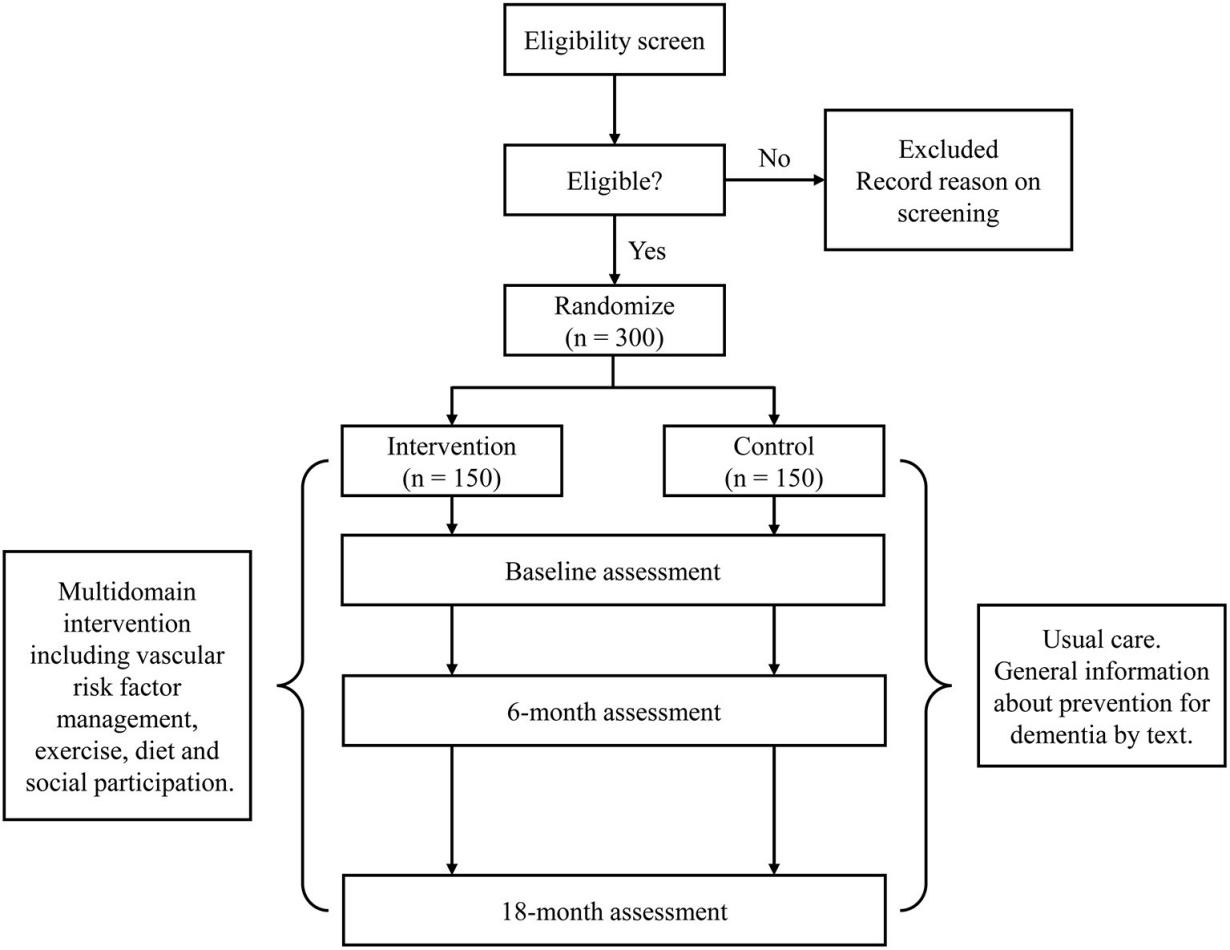
Study flow of the Japan-multi-domain intervention trial for prevention of dementia in older adults with diabetes (J-MIND-Diabetes).

The J-MIND-Diabetes study was designed as a 18-month, open-labeled, randomized, controlled, multicenter trial of multi-domain intervention targeting modifiable risk factors for dementia among T2DM patients with cognitive impairment. To be organized by a central coordinating center at the National Center for Geriatrics and Gerontology (NCGG), this study involves 16 centers located in Japan. This study will randomize 300 T2DM patients with cognitive impairment into intervention or control groups. The multi-domain intervention will address the following 4 domains: (1) management of metabolic and vascular risk factors (diabetes, hypertension, and dyslipidemia); (2) physical exercise and self-monitoring of physical activity; (3) nutritional guidance; and (4) promoting social participation. The control group will receive usual diabetes care and general instructions on dementia prevention. The primary and secondary outcomes will be assessed at baseline, 6, and 18 months in both groups (see the study flow diagram shown in Figure 1).

### Ethic Committee Review and Approval

All study procedures have been reviewed and approved by the Institutional Review Boards (IRBs) at all participating institutions, and this trial has been registered on the University Hospital Medical Information Network Clinical Trials Registry (UMIN-CTR) as number UMIN000035911. The purpose, nature, and potential risks of this trial will be fully explained to the participants, and all participants will provide written informed consent before participating in the trial.

### Eligibility Criteria (Inclusion/Exclusion Criteria)

Table 1 shows the inclusion and exclusion criteria for the J-MIND-Diabetes trial. In order to focus on those at increased risk of dementia, this trial focuses on T2DM patients who are classified into category II (mild cognitive impairment to mild dementia) based on the “Glycemic Targets for Elderly Patients with Diabetes” as they were put forth by the JDS/JGS Joint Committee (Japan Diabetes Society (JDS)/Japan Geriatrics Society (JGS) Joint Committee on Improving Care for Elderly Patients with Diabetes, 2017). Therefore, patients are found eligible for the J-MIND-Diabetes trial if they (1) are diagnosed with T2DM, (2) are aged 70-85 years at enrollment, (3) have no or mild impairment of basic activities of daily living (ADL) [Barthel index (Mahoney and Barthel, 1965) score of 80 or more], (4) are mildly cognitively impaired [the Japanese version of the Montreal Cognitive Assessment (MoCA-J) (Fujiwara et al., 2010) score of less than 26], (5) have a Mini-Mental State Examination (MMSE) (Folstein et al., 1975) score of 21-30, (6) are outpatients or those who have a stable clinical course for more than 4 weeks after their last hospitalization or institutionalization and whose medical examination classification need no change during the monitoring period, (7) are accompanied by a co-participant (study partner), and (8) provide written informed consent on their own or via their study partners.

**Table 1:** Inclusion and exclusion criteria for the J-MIND-Diabetes trial.

**Inclusion criteria**
1.Patients with type 2 diabetes mellitus
2.Patients aged 70–85 years at enrollment
3.Patients with no or mild impairment of basic activities of daily living (Barthel Index score of 80 or more)
4.Patients with a Japanese version of the Montreal Cognitive Assessment score of less than 26
5.Mini-Mental State Examination score, between 21 and 30
6.Outpatients or those exhibiting a stable clinical course for more than 4 weeks after their last hospitalization or institutionalization and whose medical examination classification need no change during the monitoring period
7.Patients accompanied by a co-participant (study partner)
8.Those who gave written informed consent
**Exclusion criteria**
1.Patients with extremely poor metabolic control (fasting plasma glucose level over 250 mg/dl; or moderate or severe urinary ketone levels).
2.Patients with new hemorrhage in the ocular fundus caused by proliferative retinopathy
3.Patients with renal failure
4.Patients with ischemic heart disease and cardiopulmonary disorders
5.Patients with bone or joint disease
6.Patients with acute infectious disease
7.Patients with diabetes gangrene.
8.Patients with severe autonomic neuropathy
9.Patients with decreased cognitive function due to Parkinson's disease, apoplexy, Huntington's disease, normal pressure hydrocephalus, brain tumors, progressive supranuclear palsy, corticobasal degeneration, multiple system atrophy, aphasia, epilepsy, subdural hematoma, encephalitis/meningitis, multiple sclerosis, or head injury
10.Patients with any local lesion, such as cerebral infarction(s) detected by CT or MRI before enrollment, that may greatly affect cognitive function
11.Patients with a history of major depression, bipolar disorder, schizophrenia, or alcohol/drug abuse; those with current serious or unstable disease
12.Patients unsuitable for treatment due to vitamin B1/B12 and/or folate deficiency, syphilis, or thyroid dysfunction
13.Patients deemed ineligible for enrollment by their responsible investigator or co-investigator at participating institutions

*J-MIND-Diabetes, japan-multi-domain intervention trial for prevention of dementia in older adults with diabetes*.

Patients are to be excluded if they have extremely poor metabolic control (fasting plasma glucose level, over 250 mg/dl; or moderate urinary ketone values) that would preclude or restrict exercise, hemorrhage in the ocular fundus caused by proliferative retinopathy, renal failure, ischemic heart disease and cardiopulmonary disorders, bone or joint disease, acute infectious disease, diabetes gangrene, and severe autonomic neuropathy (Table 1). Moreover, patients are to be excluded if they have diseases which greatly affect their cognitive function (Parkinson's disease, apoplexy, Huntington's disease, normal pressure hydrocephalus, brain tumors, progressive supranuclear palsy, corticobasal degeneration, multiple system atrophy, aphasia, epilepsy, subdural hematoma, encephalitis/meningitis, multiple sclerosis, head injury, any local lesion detected by brain computed tomography (CT) or magnetic resonance imaging (MRI), major depression, bipolar disorder, schizophrenia, alcohol/drug abuse and untreatable vitamin B1/B12 and/or folate deficiency, syphilis, or thyroid dysfunction) (Table 1).

### Enrollment and Assessment Procedures

#### Recruitment

Since T2DM outpatients who fall under category II based on the JDS/JGS Joint Committee's guidelines (Japan Diabetes Society (JDS)/Japan Geriatrics Society (JGS) Joint Committee on Improving Care for Elderly Patients with Diabetes, 2017) and have mild cognitive impairment comprise the target population, their initial eligibility will be determined based on their clinical charts by the investigators. Then, potentially eligible patients with T2DM aged 70-85 years will be assessed for cognitive function [MoCA-J (Fujiwara et al., 2010) and MMSE (Folstein et al., 1975)] and basic ADL [Barthel Index (Mahoney and Barthel, 1965)], with their final eligibility determined by the investigators and clinicians according to the inclusion and exclusion criteria.

#### Assessment

**Figure 2:**
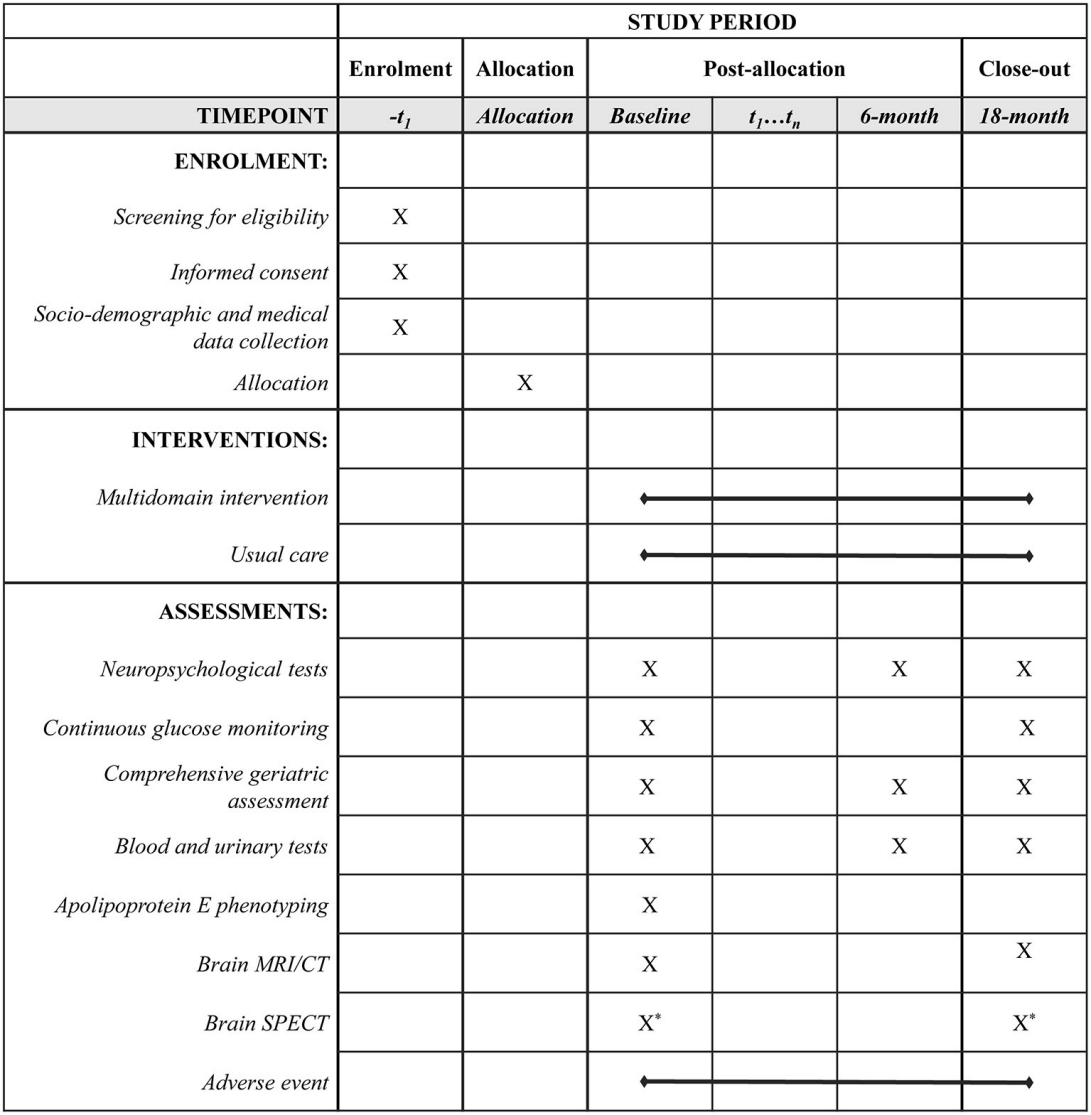
Timeline for participants in the J-MIND-Diabetes trial. ^*^: Non-mandatory, -t_1_: visit within 8-week before the day of allocation, t_1_…t_n_: visit to the hospital during the study period. CT, computed tomography; J-MIND-Diabetes, japan-multi-domain intervention trial for prevention of dementia in older adults with diabetes; MRI, magnetic resonance imaging; SPECT, single photon emission computed tomography.

All subjects will complete neuropsychological tests, comprehensive geriatric assessments (CGA), and blood and urinary tests at baseline, as well as at 6- and 18-month follow-up. Additionally, brain MRI or CT will be performed by the scanner available at each institution at baseline and 18-month follow-up to detect any local lesion such as cerebral infarctions that can greatly affect cognitive function. Adherence to the intervention programs and adverse events will be also monitored during this trial. The timeline for scheduled assessments following the SPIRIT guidelines is shown in Figure 2.

Psychologists at each institution were trained in the implementation and scoring methods of neuropsychological tests by participating in the training session held at the NCGG. Moreover, scoring for the Rey-Osterrieth Complex Figure Test (ROCFT) (Strauss et al., 2006) will be conducted by a single psychologist from the NCGG to minimize variability in scoring between neuropsychologists.

#### Primary Outcome: Cognitive Change at 18-Month Follow-Up (Composite Score)

The primary outcome is change from baseline at 18 months in a global composite score using several neuropsychological tests (Table 2), which include tests of global cognitive function [MoCA-J (27), MMSE (Folstein et al., 1975)]; memory [ROCFT (Strauss et al., 2006), delayed recall test of a 10-word list derived from the Alzheimer's Disease Assessment Scale-cognitive subscale (ADAS-Cog) (Mohs and Cohen, 1988)]; attention [Wechsler Adult Intelligence Scale (WAIS) Digit Span (Wechsler, 1997)]; executive function/processing speed [Trail Making Test (TMT) (Lezak, 2004), Digit Symbol Substitution Test (DSST) (Wechsler, 1997), Letter word fluency test (Lezak, 2004)]. The composite score will be generated by averaging the Z scores of each neuropsychological test standardized with baseline means and standard deviations (SDs) for each test from the intention-to-treat population. A Z score of −1, for example, represents a score that is 1 SD below the baseline mean. A one-point decrease on the composite score indicates an average decline of 1 SD across the neuropsychological tests.

**Table 2:** Primary outcome measures in the J-MIND-Diabetes trial.

**Domain**	**Neuropsychological test**
Global cognitive function	• Japanese version of the montreal cognitive assessment (Fujiwara et al., 2010) • Mini-mental state examination (Folstein et al., 1975)
Memory	• Rey-osterrieth complex figure test (Strauss et al., 2006) • Word-list memory [ADAS-cog (Mohs and Cohen, 1988)]
Working memory	• Digit span test [WAIS-III (Wechsler, 1997)]
Processing speed	• Digit symbol substitution test [WAIS-III (Wechsler, 1997)]
Attention	• Trail making test part A (Lezak, 2004)
Executive function	• Trail making test part B (Lezak, 2004) • Letter word fluency test (Lezak, 2004)

*ADAS-cog, alzheimer's disease assessment scale-cognitive subscale; J-MIND-Diabetes, japan-multi-domain intervention trial for prevention of dementia in older adults with diabetes; WAIS, wechsler adult intelligence scale*.

#### Secondary Outcomes

##### Cognitive Change at 6-Month Follow-Up (Composite Score) and Change in Scores of Each Neuropsychological Test at 6-/18-Month Follow-Up

Secondary outcomes related to cognitive changes are: change from baseline at 6 months in the global composite score described for the primary outcome; and change from baseline in scores of each neuropsychological test at 6-/18 month follow-up.

##### Improvement in Outcomes Related to T2DM

- Metabolic control: In blood tests performed at baseline and 6-/18-month follow-up, the participants will be assessed for glycated hemoglobin (HbA1c), glycoalbumin, fasting blood glucose levels, and fasting blood insulin levels. Additionally, at baseline and 18-month follow-up, they will also be evaluated for SD of blood glucose levels, mean amplitude of glycemic excursions (MAGE), continuous overall net glycemic action (CONGA), and mean of daily differences (MODD) (Inchiostro et al., 2013) by using the FreeStyle Libre Pro system (Abbott Diabetes Care, Tokyo, Japan), a continuous glucose monitoring (CGM) system capable of providing records of glucose levels, trends, and patterns in each individual for up to 14 days. Moreover, the averaged percentage of time each participant spent in hypoglycemia (<70 mg/dL) will be also calculated.
- Self-reported hypoglycemic events: Participants will be assessed for hypoglycemic events by asking the following questions: “Did you experience any hypoglycemia episode that required the assistance of another person in the past year?” (Yes/No); “Did you experience any symptoms, such as sweating, palpitation, or trembling, in the past year?” (Yes/No); and “Did you experience any symptoms, such as lightheadedness, unsteadiness, dizziness, or visual disturbance, in the past year?”
- Microangiopathy and macroangiopathy: Participants will be assessed at baseline and at follow-up for microangiopathy including retinopathy, nephropathy, and neuropathy. Participants will be assessed for retinopathy categorized into five stages (Wilkinson et al., 2003): (1) no apparent retinopathy, (2) mild non-proliferative retinopathy, (3) moderate non-proliferative retinopathy, (4) severe non-proliferative retinopathy, (5) proliferative diabetic retinopathy. They will also be assessed for presence or absence of edema in the macula of the retina as well as for visual acuity by the Landolt Ring Test. Participants will be assessed for diabetic nephropathy categorized into five stages based on urinary albumin (mg/gCr), urinary protein (g/gCr), or estimated glomerular filtration rate (eGFR, mL/min/1.73m2) (Japan Diabetes Society, 2016): (1) Pre-nephropathy, (2) incipient nephropathy, (3) overt nephropathy, (4) kidney failure, (5) dialysis therapy. They will also be assessed for diabetic neuropathy using the Achilles tendon reflex test and vibration testing with a 128-Hz graduated tuning fork. Again, they will also be assessed for macroangiopathy, including ischemic heart disease, stroke, and peripheral vascular disease, based on their clinical charts and MR or CT imaging (Japan Diabetes Society, 2016).

##### Improvement in Parameters as Part of Comprehensive Geriatric Assessment (CGA)

CGA includes following specific measurements:

- Basic ADL: Participants will be evaluated for basic ADL using the Barthel index (Mahoney and Barthel, 1965), which includes 10 items to assess basic self-care abilities, such as feeding, transfer from bed to chair, bathing, bowel control, and bladder control, where the score ranges from 0 (complete dependence) to 100 (complete independence).
- Instrumental ADL: Participants will also be assessed for instrumental ADL using the Lawton index (Lawton and Brody, 1969), which includes 8 items, such as using the telephone, shopping, and handling medication, where the score ranges from 0 (low function) to 8 (high function).
- Depressive symptoms: Participants will be evaluated for depressive symptoms using the 15-item Geriatric Depression Scale (Yesavage et al., 1982), where the score ranges from 0 to 15, with higher scores indicating more depressed mood.
- Physical performance: For all participants, usual gait speed over a distance of 2.4 m at the mid-point of the walkway that has both acceleration and deceleration zones (1 m, respectively) will be measured twice and a mean value will be calculated (Guralnik et al., 1994). Both right- and left-hand grip strengths will be measured using a standard digital hand grip dynamometer (Takei Scientific Instruments Co., Ltd, Japan) at standing position with shoulder adducted and neutrally rotated and elbow in full extension (Watanabe et al., 2005). One-leg standing tests will also be performed on all participants (Michikawa et al., 2009) using the one-leg standing test to measure how long (in seconds) each participant in bare may be able to stand unassisted on one leg as long as possible with eyes open for up to 60 s, and an average of left and right one-leg standing times will be calculated.
- Physical frailty and sarcopenia: Physical frailty will be defined for all participants based on the frailty phenotype proposed by Fried et al. in the Cardiovascular Health Study (Fried et al., 2001), which consists of shrinking, weakness, slowness, self-reported exhaustion, and low physical activity. In addition, to determine the presence or absence of sarcopenia (Chen et al., 2014; Cruz-Jentoft et al., 2019), appendicular muscle mass (AMM) will be measured in all participants by bioelectrical impedance analysis, and skeletal muscle mass index will be calculated as AMM divided by height squared (kg/m2).
- History of falls and fall risk: All participants will also be examined for any history of falls within the past 12 months. The Fall Risk Index (Kikuchi et al., 2009) consisting of 21 questions, including the following 3 subcategories, physical function (8 items), geriatric syndrome (8 items) and environmental hazards (5 items) will be used to detect their risk of falls, where each item receives a score of 1 (risk present) or 0 (risk absent), with a higher sum of scores indicating a higher risk of falls.
- Nutritional status: All participants will be assessed for their nutritional status using the Mini-Nutritional Assessment Short-Form (Rubenstein et al., 2001) composed of 6 questions, where the score ranges from 0 to 14, with a higher score indicating better nutritional status.
- Intakes of nutrients and foods: All participants will be assessed at baseline and at 18-month follow-up with the Food Frequency Questionnaire (Sasaki et al., 2003), which is designed to assess the average intake of 47 foods and beverage to estimate their daily food-group consumption and nutrient intakes.
- Appetite: Participants will also be assessed for their appetite using the Simplified Nutritional Appetite Questionnaire (Nakatsu et al., 2015), where the total score ranges from 4 to 20, with a higher score indicating a better appetite.
- Social network: The Lubben Social Network Scale 6 (Lubben et al., 2006) composed of 6 questions will be used to assess each participant's social network, where the score ranges from 0 to 30, with a higher score indicating better social network.
- Social participation: Participants will also be assessed for their social participation by a questioner about their social participation in eight types of groups (Kanamori et al., 2014): (1) neighborhood associations/senior citizen clubs/ fire-fighting teams (Local Community); (2) hobby groups (Hobby); (3) sports groups or clubs (Sports); (4) political organizations or groups (Politics); (5) industrial or trade associations (Industry); (6) religious organizations or groups (Religion); (7) volunteer groups (Volunteer); and (8) Others.

##### Improvement in Blood and Urinary Markers

Blood and urinary markers assessed in all participants at baseline and follow-up will include total protein, albumin, aspartate aminotransferase, alanine aminotransferase, γ-glutamyl transpeptidase, total cholesterol, high density lipoprotein-cholesterol, triglyceride, creatinine, eGFR, blood urea nitrogen, sodium, potassium, chloride, calcium, phosphorus, free triiodothyronine, free thyroxine, thyroid stimulating hormone, rapid plasma reagin, treponema pallidum hemagglutination, brain natriuretic peptide, vitamin B1, vitamin B12, folate, urine glucose, urine protein, occult blood in urine, and u-Alb/Cr. In addition, all participants will be assessed at baseline for their apolipoprotein E phenotype.

#### Adverse Events and Serious Adverse Events

To evaluate the safety of the intervention, all adverse events and serious adverse events will be monitored during this trial. Information to be collected about adverse events will include their date of onset, severity, associated treatment, consequences, and causation. Serious adverse events will be reported to the principal investigator, IRB, and co-researchers immediately.

### Interventions Procedures

#### Intervention Arm

The intervention group will participate in multi-domain intervention programs intended to promote: (1) management of metabolic and vascular risk factors; (2) physical exercise and self-monitoring of physical activity; (3) nutritional guidance; and (4) social participation.

##### Management of Metabolic and Vascular Risk Factors

While participants will be treated for T2DM, hypertension, and dyslipidemia in accordance with clinical guideline recommendations in Japan, they will be treated for T2DM based on the Treatment Guidelines for Elderly Patients with Diabetes Mellitus 2017 issued by the JDS/JGS Joint Committee (Japan Diabetes Society (JDS)/Japan Geriatrics Society (JGS) Joint Committee on Improving Care for Elderly Patients with Diabetes, 2017), which recommend that the glycemic target (HbA1c) be determined for each patient by taking into account his or her background characteristics and health status and classifying the patient into 1 of the following 3 categories: category I (intact cognitive function and ADL), category II (mild cognitive impairment to mild dementia or impairment of instrumental ADL), and category III (moderate or severe dementia, impairment of basic ADL, or presence of multiple comorbidities). As a specific feature of these categories, the lower limit of each glycemic target is specified for those receiving drugs associated with high risk of severe hypoglycemia, for example, insulin, sulfonylureas, and glinides. In this trial focused on subjects classified as category II, therefore, the upper limit of the glycemic target is to be set at 7.0% for patients not receiving these drugs, while the upper and lower limits of the glycemic target are to be set at 8.0% and 7.0%, respectively, for those receiving these drugs. Additionally, those in the intervention group will undergo CGM assessments to avoid hypoglycemia and glycemic fluctuations; while, likewise, those in the control group will undergo CGM assessments, clinicians will be blinded to the results of CGM assessments in these patients.

##### Physical Exercise and Self-Monitoring of Physical Activity

All participants will engage at least twice a month in a physical exercise program provided at participating institutions by study physiotherapists or trained instructors, which consists of aerobic exercise, muscle strength training, postural balance retraining, and dual-task training, namely “cognicise” (“cogni” for cognition + “cise” for exercise), involving physical and cognitive tasks. The combined activity program intervention including cognicise was shown to improve cognitive and physical performance in older adults with MCI (Shimada et al., 2018). This program will be composed of a 5-min medical check (blood pressure, pulse rate), a 15- to 20-min training including stretching, muscle strength exercise and postural balance training, a 5-min rest, a 20- to 30-min aerobic exercise, a 5-min rest, and a 20- to 30-min dual-task training. The aerobic exercises, including stair stepping and endurance walking will be performed with the mean aerobic exercise intensity set as moderate intensity according to the Karvonen formula: Target heart rate [HR] = [40–60% × (maximum HR – resting HR)] + resting HR. The maximum HR will be estimated for each participant by using the age-predicted maximal HR formula (220 – age). All participants will be assessed for HR immediately after an aerobic exercise based on their pulse rates. The participants will also be recommended to conduct daily home-based muscle-strengthening exercises, walking, and dual-task training at least twice a week, as well as to self-monitor these activities using study-provided booklets and pedometers.

##### Nutritional Guidance

Based on the JDS/JGS Joint Committee guidelines (Japan Diabetes Society (JDS)/Japan Geriatrics Society (JGS) Joint Committee on Improving Care for Elderly Patients with Diabetes, 2017), nutritional counseling sessions will be conducted individually by registered dieticians at each medical examination (performed approximately once every two months). Nutritional counseling for the participants include guidance on (1) appropriate energy intake (total energy, carbohydrate, protein, lipid, fiber, salt) to improve their glycemic control and cognitive and physical condition including frailty and sarcopenia; (2) improvement of lifestyle and dietary behavior through smoking cessation and reduced alcohol consumption; (3) intake of dietary staples (cooked rice, bread, or noodles), main dishes (fish, chicken, soybeans and soybean products), and side dishes (vegetables and seaweeds) based on the place mat to increase dietary diversity; and (4) chewing and swallowing function and oral care.

**Figure 3:**
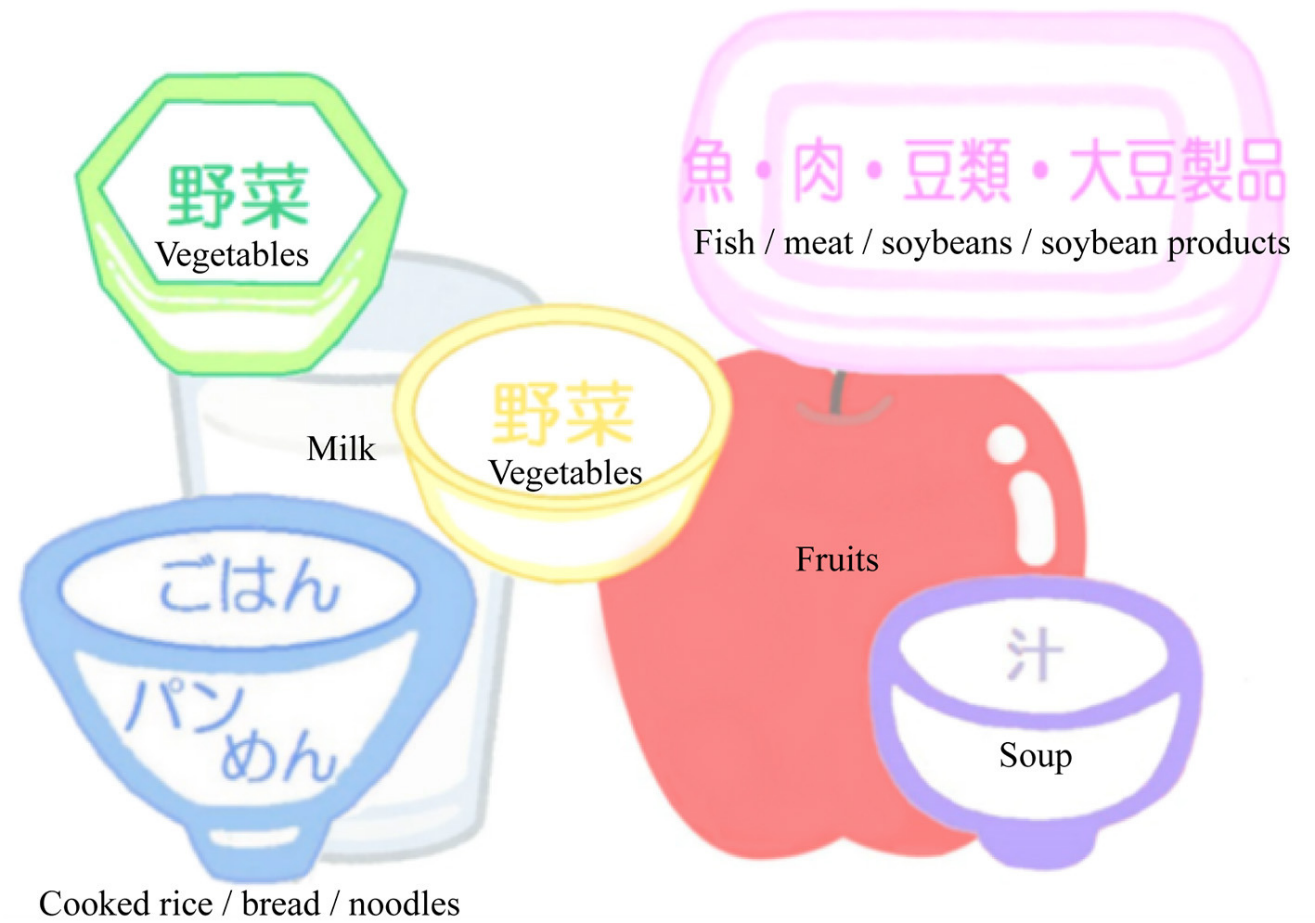
Place mat illustrating dietary variety. Subjects are instructed to take well-balanced diet and increase dietary diversity arranging dietary staples (Cooked rice, bread, or noodles), main dishes (fish, meat, soybeans and soybean products), side dishes (green and yellow vegetables and seaweeds), soup, fruits, and milk and dairy products on a place mat.

Additionally, participants will be requested to monitor their body weight, start of meal times, and dietary diversity every day using a study-provided place mat illustrating dietary variety (Figure 3) and a dietary diary. Participants will be instructed to take well balanced diet and increase dietary diversity arranging dietary staples, main dishes, side dishes, soup, fruits, and milk and dairy products on a study-provided place mat. Moreover, dietary diary will be reviewed by dieticians in nutritional counseling session, and their feedback will be given to the participants. Dieticians will also provide two kinds of foods, which include: rice with mixed grains to increase fiber intake; canned mackerel, salmon, and sardine to increase intake of proteins, eicosapentaenoic acid (EPA), and docosahexaenoic acid (DHA); fish sausage to increase intake of proteins and calcium, and cooking recipes at every counseling session.

##### Social Participation

To promote social participation, participants will be requested to go out at least three times a week. Moreover, participants will be requested to monitor their social activities including taking care their grand-children, doing work/volunteer work, face-to-face conversation, shopping/eating-out, and participating in organizations or groups by using a study-provided booklet every day. Again, to encourage an active lifestyle, a meeting will be heled after each physical exercise session, where participants discuss their physical, social, and cognitive activities among themselves.

#### Control Group

Participants in the control group will receive usual care and treatment for T2DM based on the Treatment Guidelines for Elderly Patients with Diabetes Mellitus 2017 as in the intervention group. Additionally, at baseline, they will receive general instructions on prevention of dementia including the benefits of physical activities, healthy diet, cognitive activities, and social participation.

### Statistical Considerations

#### Sample Size

Using the open source sample size calculator (http://www.openepi.com/SampleSize/SSMean.htm), the sample size was calculated based on preliminary data from MMSE assessments over 1-year (1.03 ± 0.36 years) in 83 patients with MCI and T2DM classified into category II among the Memory Clinic at the NCGG, since no multi-domain intervention trials have been conducted to date in this population. The mean change in MMSE score among 83 patients was −1.45 ± 3.55. Of these, the optimally controlled group (n = 30) showed a mean change in MMSE score of −0.33 ± 2.58. Based on these preliminary data, it was hypothesized that the present study is likely to detect differences in change of cognitive function between the intervention and control groups (intervention group vs. control group, −0.33 ± 2.58 vs. −1.45 ± 3.55). With a two-sided significance level of 5% and a statistical power of 80%, the total sample size required for the unpaired *t*-test was calculated as 242. In addition, dropout rates at final follow-up is estimated to be 15–20% at each institution, thus a total of 300 patients are required.

#### Randomization and Blinding

In this open-label intervention study, participants will be centrally randomized at a 1:1 allocation ratio into intervention and control groups using the stratified permuted-block randomization methods based on the following stratification variables:

1. Institutions (1–15 institutions).
2. Age at enrollment (70–77 years vs. 78–85 years).
3. Glycemic control relative to the glycemic targets recommended by the JDS/JGS joint committee (optimally controlled vs. poorly or excessively controlled).
4. Drugs potentially associated with severe hypoglycemia such as insulin, sulfonylureas and glinides (those receiving these drugs vs. those not receiving these drugs).

The randomization sequences will be generated by a trial statistician (H. N.) by using Proc Plan in SAS version 9.4 (SAS Institute, Cary, NC, USA). Study participants and research staff including investigators and clinicians at each institution will be unaware of the randomization sequences and block size. Research staff responsible for primary outcome data collection will also be blinded to group assignment, to which, however, the intervening research staff and the participants will be not blinded.

#### Data Collection Forms and Data Monitoring

All measurements will be assessed by trained personnel. Most outcome measurements will be collected using papers forms at each institution. Then, these data will be collected through entry of these data by assessors using electronic data capture (EDC) systems. Paper forms will be retained as back-ups at each institution as required.

On-site monitoring will be conducted at each institution to ensure that the patient rights are protected, the reported data are accurate, and the conduct of the trial is compliant with the current approved protocol. The monitor will ensure that (1) written informed consent is obtained from all participants before their participation in this trial; (2) primary outcome data reported in the EDC are complete and accurate; and (3) all adverse events and serious adverse events are reported appropriately.

#### Data Analyses

Data will be presented as means, medians, standard deviations, ranges and interquartile ranges for continuous and ordinal variables, and counts and percentages for categorical variables. Differences in baseline clinical characteristics between the intervention and control groups will be examined for significance by using the *t*-test or the Mann-Whitney U test for continuous variables and by the chi-squared test or the Fisher's exact test for categorical variables, as appropriate.

To evaluate differences in cognitive changes from baseline at 18-month follow-up between the intervention and control groups, the mixed-effects model for repeated measures (MMRM) with an unstructured covariance structure will be conducted using groups, time of the visit, group by time interaction, and baseline composite cognitive score as covariates. For the secondary continuous variables, the same analyses using MMRM will be performed. For the secondary categorical variables, logistic regression analyses or chi-squared tests will be conducted as appropriate. Frequencies of adverse events and serious adverse events will be summarized and compared between the intervention and control groups by using the chi-squared test or the Fisher's exact test.

All statistical analyses will be performed using SAS 9.4 (SAS Institute, Cary, NC, USA). *P*-values <0.05 will be considered statistically significant.

## Discussion

To date, no multi-domain intervention trial has been conducted in older adults with T2DM for prevention of cognitive decline and dementia, although this population is at higher risk of cognitive decline and dementia. The J-MIND-Diabetes trial will thus be the first to demonstrate the effectiveness of multi-domain intervention in this population and help establish effective preventive strategies for cognitive decline and dementia.

## Ethics Statement

The studies involving human participants were reviewed and approved by National Center for Geriatrics and Gerontology. The patients/participants provided their written informed consent to participate in this study.

## Author Contributions

TSa obtained funding for the trial. AA, NI, TI, JK, MK, KKob, KKou, MO, NS-A, KS, SS, YTak, YTam, HU, HW, and YY obtained ethical approval. AA, HF, NI, TI, JK, MK, KKob, KKou, MO, NS-A, SS, YTak, YTam, HT, HU, HW, and YY recruited subjects. KH and HS provided comments on intervention procedures. HN provided comments on statistical analysis and generated randomization sequences. TSu and TSa wrote the main manuscript text. All authors made substantial contributions to the conception, study design, and read and approved the final version of the manuscript.

## Conflict of Interest

AA has received speaker honoraria from Merck Sharp and Dohme, Dainippon Sumitomo Parma Co., Tanabe Mitsubishi Pharma Corporation, Kyowa Hakko Kirin Co. Ltd., and Takeda Pharmaceutical Co. Ltd. NI has received honoraria from Abbott Japan LLC. The remaining authors declare that the research was conducted in the absence of any commercial or financial relationships that could be construed as a potential conflict of interest.
